# Association of vision impairment and blindness with socioeconomic status in adults 50 years and older from Alto Amazonas, Peru

**DOI:** 10.1038/s41433-021-01870-x

**Published:** 2022-02-03

**Authors:** John M. Nesemann, Noelia Morocho-Alburqueque, Alvaro Quincho-Lopez, Marleny Muñoz, Sandra Liliana-Talero, Emma M. Harding-Esch, Martha Idalí Saboyá-Díaz, Harvy A. Honorio-Morales, Salomón Durand, Cristiam A. Carey-Angeles, Jeffrey D. Klausner, Andres G. Lescano, Jeremy D. Keenan

**Affiliations:** 1 Francis I. Proctor Foundation, University of California, San Francisco, CA USA; 2 David Geffen School of Medicine, University of California, Los Angeles, CA USA; 3 Emerge, Emerging Diseases and Climate Change Research Unit, School of Public Health and Administration, Universidad Peruana Cayetano Heredia, Lima, Peru; 4 Universidad Nacional de Piura, Piura, Peru; 5 Universidad Nacional Mayor de San Marcos, Lima, Peru; 6 Área de Epidemiología, Red de Salud Alto Amazonas, Yurimaguas, Peru; 7 Escuela Superior de Oftalmología del Instituto Barraquer de América, Bogotá, Colombia; 8 Clinical Research Department, London School of Hygiene & Tropical Medicine, London, UK; 9 Department of Communicable Diseases and Environmental Determinants of Health, Pan American Health Organization, Washington, DC USA; 10 Componente de Salud Ocular y Prevención de la Ceguera, Ministerio de Salud, Lima, Peru; 11 Área de Epidemiología, Dirección Regional de Salud Loreto, Iquitos, Peru; 12 Universidad Nacional de la Amazonia Peruana, Iquitos, Peru; 13 Department of Population and Public Health Sciences, Keck School of Medicine, University of Southern California, Los Angeles, CA USA; 14 Department of Ophthalmology, University of California, San Francisco, CA USA

**Keywords:** Epidemiology, Risk factors, Vision disorders, Epidemiology

## Abstract

**Objective:**

To determine the relationship between socioeconomic status (SES) and visual impairment (VI) or blindness in the rural Peruvian Amazon, hypothesizing that higher SES would have a protective effect on the odds of VI or blindness.

**Methods:**

In this cross-sectional study of 16 rural communities in the Peruvian Amazon, consenting adults aged ≥ 50 years were recruited from ~30 randomly selected households per village. Each household was administered a questionnaire and had a SES score constructed using principal components analysis. Blindness and VI were determined using a ministry of health 3-meter visual acuity card.

**Results:**

Overall, 207 adults aged ≥ 50 were eligible; 146 (70.5%) completed visual acuity screening and answered the questionnaire. Of those 146 participants who completed presenting visual acuity screening, 57 (39.0%, 95% CI 30.2–47.1) were classified as visually impaired and 6 (4.1%, 95% CI 0.9–7.3) as blind. Belonging to the highest SES tercile had a protective effect on VI or blindness (OR 0.29, 95% CI 0.09 to 0.91, *p* = 0.034), with a linear trend across decreasing levels of SES (*p* = 0.019). This observed effect remained significant regardless of how SES groups were assigned.

**Conclusion:**

Belonging to a higher SES group resulted in a lower odds of VI or blindness compared to those in the lowest SES group. The observation of a dose response provides confidence in the observed association, but causality remains unclear. Blindness prevention programs could maximize impact by designing activities that specifically target people with lower SES.

## Introduction

Overall, 405 million people worldwide are estimated to live with visual impairment (VI), 76% of whom suffer from a treatable or preventable cause, with the majority living in low-income countries with minimal access to detection and treatment [1, 2]. Socioeconomic status (SES) has been associated with a variety of adverse visual outcomes, including VI [3, 4], and this relationship is accentuated in rural areas [5, 6]. While some studies on the prevalence of VI and its associated risk factors have been conducted in the Brazilian Amazon, few studies exist for Peru and fewer still have examined the association between SES and VI [7–9]. Furthermore, many of the existing studies failed to use a composite, asset-based measure of SES. Low SES might be expected to have a particular impact on health outcomes in the Peruvian Amazon, given the remoteness of communities and relative inaccessibility of eye care. In this study, we sought to determine the relationship between SES and VI in the Alto Amazonas region of Peru, hypothesizing that higher SES would have a protective effect on the odds of VI and blindness.

## Materials and methods

### Study design and setting

Peru is organized into 26 regions, which are further subdivided into provinces and districts. Alto Amazonas is one of eight provinces in the region of Loreto, covering 18,764 km^2^ and containing a population of 122,725 in its six districts (i.e., Balsapuerto, Lagunas, Santa Cruz, Jeberos, Teniente César López Rojas, and Yurimaguas) [10]. Supplementary Fig. 1 depicts the study location within Peru and the location of study villages, other villages, and optometrists or ophthalmologists offices. According to the 2017 census, 83,584 (68.1%) of the total population was urban and 12,756 (15.1%) of those 12 years old and above identified as indigenous [10]. A 2018 report by Peru’s National Institute of Statistics and Informatics found that out of the 1,874 districts in Peru, Balsapuerto ranked as the 249th poorest district, Lagunas as the 578th, Santa Cruz as the 580th, Jeberos as the 878th, Teniente César López Rojas as the 978th, and Yurimaguas as the 1031st. The proportion of the population living below the poverty line ranged from 44.3 to 62.5% in Balsapuerto and 27.6 to 35.9% in Yurimaguas. For comparison, the Iquitos district, containing the capital city of the Loreto region, was ranked 1752nd with 6.7–10.6% of the population below the poverty line while the Miraflores district in Lima was ranked 1873rd with 0.0–0.2% of the population below the poverty line [11]. In this cross-sectional study, 22 communities, defined as settlements of 100 people or more [12], in the Alto Amazonas region of Peru were randomly selected from a sampling frame of 105 communities for participation in a trachoma prevalence survey using probability-proportional-to-size sampling after excluding urban areas (i.e., the capital city of each district and the district of Yurimaguas, given the expected low burden of trachoma in urban areas) [13, 14]. In addition to collecting data on trachoma, visual acuity in adults 50 years and older was measured in order to leverage the effort necessary to reach these areas. After field testing the visual acuity procedures in several communities during the first round of fieldwork, the subsequent 16 communities were selected for this sub-study. Within each village, ~30 randomly selected households were visited and all adults 50 years and older within the household invited to participate. Fieldwork was conducted from January to March of 2021.

The sample size was based on the underlying trachoma prevalence survey and therefore fixed. Assuming (1) 30 households and 180 people aged ≥ 1 year would be surveyed per community, (2) 10% of the population would be ≥50 years (i.e., ~288 people across all communities, and thus 96 per tercile), (3) the prevalence of VI and blindness would be ~20% [9], and (4) an alpha of 0.05, then the study would provide ~80% power to estimate a 15% or greater difference in the prevalence of blindness or VI between the lowest and highest SES terciles (i.e., 10% vs 25% VI).

### Data collection

Vision was tested in a central location in the village by field workers who had undergone a 1-week training using a Ministry of Health endorsed 3-meter visual acuity card (Ministerio de Salud, Lima, Peru). The card consists of six lines of tumbling E optotypes of different sizes (corresponding to 20/200, 20/100, 20/70, 20/50, 20/40, and 20/30) (Supplementary Fig. 2). Individuals were seated 3 meters away from the card in ambient lighting conditions and each eye was tested separately. A successful effort required the correct identification of half or more of the optotypes in each line. If participants were unable to detect the optotypes, the fieldworker tested them for the ability to read the largest optotype at 1.5 m (i.e., 20/400), counting fingers (CF) at 1.5 m, hand motion (HM) at 1.5 m, or light perception (LP) at 30 cm. If the participants could not perceive light they were recorded as no LP (NLP). Participants were first tested with spectacle correction if available (i.e., the World Health Organization [WHO]’s definition of presenting visual acuity), followed by pinhole occlusion over any correction.

Each head of household was administered a socioeconomic survey, with survey items modified from the questions from Peru’s 2012 Demographic and Health Survey, with input from local health workers and researchers [15]. Since the study was planned for a rural and relatively resource-limited population, the questionnaire focused on asset-based measures to capture SES instead of information on consumption, expenditure, or income [16, 17].

### Definitions and conventions

Visual acuity for the better seeing eye was used in all analyses, categorized according to the WHO’s International Classification of Disease [18]. Individuals with visual acuity worse than 20/60 up to 20/400 were considered visually impaired, with individuals scoring 20/70, 20/100, and 20/200 considered moderately visually impaired, and those scoring 20/400 considered severely visually impaired. Those scoring worse than 20/400 (i.e., CF, HM, LP, NLP) were considered blind.

A principal components analysis (PCA) was used to construct a SES index for each household. As PCA works best when asset variables exhibit varied distribution across households, assets owned by all or no households were removed. Multilevel categorical SES variables were dichotomized (e.g., type of floor converted into presence or absence of wooden floor, dirt floor, or brick floor). The statistical methods used to convert survey questions into a SES score have been described elsewhere [19]. The PCA in this study was conducted via singular value decomposition of the centered and scaled data-matrix in order to account for differences in the units of measurement for each variable (e.g., quantitative variables like number of birds were given equal weight as binary ownership variables). The first principal component score for each household was assumed to be a measure of SES and was standardized to a mean of 0 and standard deviation of 1. The households were then grouped into socioeconomic terciles, assigning the top third of households to the highest tercile, the lowest third to the lowest tercile, and the remaining third to the middle tercile. Other thresholds for grouping SES were explored in sensitivity analyses.

### Statistical considerations

The exposure of interest was socioeconomic tercile and the outcome was presenting VI or blindness. The relationship between SES tercile and presenting VI or blindness was assessed with an age- and sex-adjusted mixed effects logistic regression model with a random intercept for community to account for community-level clustering. A similar model was constructed for pinhole VI (i.e., VI or blindness in the better seeing eye with pinhole occlusion). Age was treated as a continuous variable in all models. Missing SES data from 39 participants, most of whom were missing only one (*N* = 19) or two (*N* = 9) fields, were imputed using the data interpolating empirical orthogonal functions approach, which has been described elsewhere [20]. In sensitivity analyses, prevalence ratios (PR) were calculated using a modified Poisson approach with robust standard errors [21]. A significance level of 0.05 was chosen for all analyses given the exploratory nature of this observational study. All analyses were performed with R version 3.6.0 (R Foundation for Statistical Computing, Vienna, Austria).

### Ethics

The study adhered to the guidelines of the Declaration of Helsinki and received ethical approval from the University of California San Francisco (reference number: 247252), Universidad Peruana Cayetano Heredia (reference number: 104344), and the Pan American Health Organization (reference number: PAHOERC.0145.03). Written informed consent was obtained for all participants; no stipend was provided.

## Results

Overall, 207 adults ≥ 50 years were eligible for inclusion; 146 (70.5%) completed visual acuity screening and answered the SES questionnaire. There was no evidence of differences between participants and non-participants in terms of sex or age (*p* value for sex = 0.848; *p* value for age = 0.640). Of the 146 participants who completed presenting visual acuity screening, 57 (39.0%, 95% CI 30.2–47.1) were classified as visually impaired (*N* = 54 with moderate VI [i.e., visual acuity of 20/70, 20/100, and 20/200 in the better seeing eye] and *N* = 3 with severe VI [i.e., visual acuity of 20/400 in the better seeing eye]) and 6 (4.1%, 95% CI 0.9–7.3) as blind (i.e., visual acuity worse than 20/400 in the better seeing eye). Of the 146 participants who completed pinhole visual acuity screening, 40 (27.4%, 95% CI 19.3–34.6) were visually impaired (*N* = 38 with moderate VI and N = 2 with severe VI) and 6 (4.1%, 95% CI 0.9–7.3%) blind. Aggregated results for each of the survey questions after dichotomization are provided in Supplementary Table 1.

**Fig. 1:**
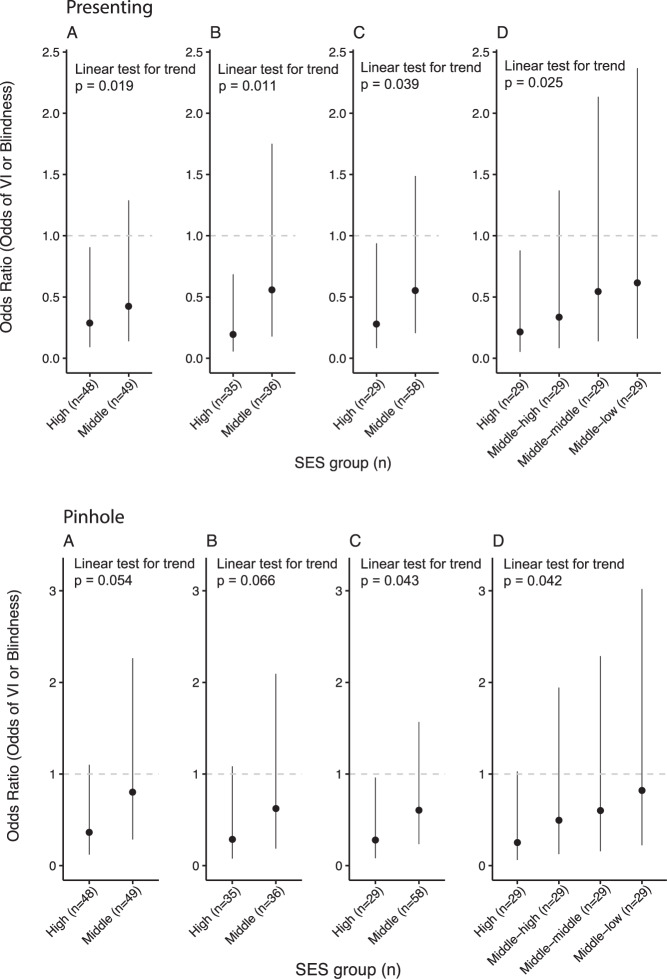
Odds ratios for presenting (first row) and pinhole (second row) visual impairment and blindness for different SES groupings. Individuals were grouped into **A** even SES terciles with imputed data, **B** SES terciles using original data, **C** uneven thirds (i.e., 20% in the highest and 40% in the middle and bottom groups) using imputed data, and **D** even quintiles (i.e., 20% in each group) using imputed data. Odd ratios were calculated relative to the lowest tercile. Vertical lines represent 95% confidence intervals and the dashed horizontal line represents an odds ratio of 1.

When categorized into terciles, the middle and lowest SES terciles had a higher percentage of individuals with both presenting and pinhole VI compared to the highest tercile (Table 1). The results of the age- and sex-adjusted mixed-effect logistic regression models for presenting VI and blindness are depicted in Fig. 1. The model found that those in the highest SES tercile had 0.29 the odds of being blind or visually impaired relative to those in the lowest SES tercile (95% CI 0.09–0.91, *p* = 0.034). Those in the middle SES tercile also had lower odds of blindness or VI compared to the lowest SES tercile, although the relationship did not achieve predetermined statistical significance (OR 0.42, 95% CI 0.14–1.29, *p* = 0.130). Conclusions did not change when analyses were repeated without imputing missing variables (OR 0.19 comparing highest to lowest SES tercile, 95% CI 0.06–0.67, *p* = 0.011).

**Table 1:** Demographic data and number blind or visually impaired in each socioeconomic tercile.

	Highest SES	Middle SES	Lowest SES
Total in group	48	49	49
Number female (%)	22 (45.8%)	23 (46.9%)	17 (34.7%)
Median age (IQR)	60 (54–68)	64 (52–69)	54 (52–64)
Presenting visual acuity			
No VI or blind (%)	31 (64.6%)	26 (53.1%)	26 (53.1%)
Number VI (%)	17 (35.4%)	21 (42.9%)	19 (38. 8%)
Number blind (%)	0 (0.0%)	2 (4.1%)	4 (8.2%)
Number VI or blind (%)	17 (35.4%)	23 (46.9%)	23 (46.9%)
Pinhole visual acuity			
No VI or blind (%)	37 (77.1%)	30 (61.2%)	33 (67.4%)
Number VI (%)	11 (22.9%)	17 (34.7%)	12 (24.5%)
Number blind (%)	0 (0.0%)	2 (4.1%)	4 (8.2%)
Number VI or blind (%)	11 (22.9%)	19 (38.8%)	16 (32.7%)

*VI* visual impairment.

Sensitivity analyses found a similar relationship between SES and VI using other common thresholds to segment individuals into SES groups [19, 22, 23]. Specifically, similar regression models were constructed with socioeconomic groups assigned as: (1) unequal thirds (i.e., 20% in the highest and 40% in the middle and bottom groups) and (2) equal quintiles (i.e., 20% in each group). The highest SES group had a lower odds of VI or blindness in both unequal SES thirds (OR 0.28, 95% CI 0.08–0.94; *p* = 0.039) and equal SES quintiles (OR 0.22, 95% CI 0.05–0.88; *p* = 0.033) relative to the lowest SES group (Fig. 1). In all cases, statistical analyses only provided strong evidence of a reduced odds in the highest SES group relative to the lowest, although evidence of a linear trend was found when comparing the odds ratios across increasing levels of SES (Fig. 1; *p* value for first-order orthogonal polynomial contrast = 0.019 using imputed data and equal SES terciles, *p* = 0.011 using complete data, *p* = 0.039 using uneven SES thirds, and *p* = 0.025 using SES quintiles). A sensitivity analysis with a modified Poisson regression was consistent with the main results (PR for highest equal tercile relative to lowest: 0.56, 95% CI 0.38–0.85; *p* = 0.006).

The results of age- and sex-adjusted mixed effects logistic regression models for the pinhole vision outcomes were consistent with the main analysis, with a similar magnitude of effect but wider confidence intervals and less statistical evidence to support the observed odds ratios and linear trends (Fig. 1).

## Discussion

This study’s principal finding was that individuals of higher SES had lower odds of presenting VI or blindness relative to the lowest SES group. This effect remained significant regardless of the thresholds used to segment individuals into SES groups and exhibited a linear dose response, thus strengthening the likelihood of the observed association.

We found that approximately 40% of individuals were classified as VI and 4% as blind when using presenting visual acuity. While the present study’s estimates have relatively wide confidence intervals, the observed prevalence of blindness was similar to a 2005 study in rural northern Peru as well as a 2019 study in the Brazilian Amazon [9, 24]. In contrast, the overall prevalence of blindness in Peru is ~2% based on a 2014 nationwide survey [25]. Estimates of VI in the present study were generally higher than those of previous studies in Peru and other parts of Latin America [9, 24–32]. It is plausible that the more rural and remote areas of Peru, such as this remote part of the Amazon, have a higher burden of VI and blindness given poorer access to ophthalmic care and fewer government –sponsored vision programs.

While we did not investigate the etiology of blindness or VI in our study population, the decrease in the proportion of individuals with presenting VI after pinhole correction from 39.0 to 24.7% suggests a relatively high burden of uncorrected refractive error in this population, similar to what has been reported in other parts of Latin America [33–35]. Pinhole correction did not change the proportion of blind individuals in the present study and more detailed evaluation of the etiology of blindness in the region is necessary. Interestingly the magnitude of the effect of SES on pinhole VI or blindness was similar to the main analysis; the wider confidence intervals and weaker statistical evidence to support the observed odds ratios are likely due to the presence of fewer visually impaired individuals after pinhole correction.

Several studies have looked at the association between VI or blindness and markers of SES. Rius et al. [4] found that illiterate, disabled, and unemployed individuals had significantly higher odds of VI in El Salvador. An ecological analysis of cross-sectional eye health surveys from seven Latin American countries found a higher prevalence of blindness and moderate VI among the more socially disadvantaged countries, determined as a composite of educational achievement, literacy, and wealth [34]. Our results agree with these prior studies and increase the rigor of the analysis through the use of household-level composite SES scores and individual analysis. It is notable that the present study found SES to be correlated with VI even within one the poorest regions of the country, where the differences between rich and poor may not be as stark as in other places. While level of education could have been used as a proxy for SES, the vast majority (84%) of adults in the area had completed primary or secondary schooling and thus educational achievement would likely have been less able to classify individuals into different groups compared to the use of a composite SES score [10]. These studies draw attention to the need for interventions to reduce blindness and VI in the most disadvantaged groups, among whom the burden is highest.

This study is relevant for public health planners in Peru and other countries with very remote populations, since it highlights that VI and blindness appear to be most common in the very population that has the least financial resources available for diagnosis or treatment. Given the expense and time required to even reach one of these villages in the Amazon, a blindness prevention program may want to design activities that specifically target those with low SES, who are both most likely to benefit from an intervention and also least likely to be able to afford an intervention on their own. Although specific activities would undoubtedly need to be adapted to the local context, examples of such interventions include offering discounted or free spectacles and cataract surgical services, providing education regarding eye diseases and available eye care services, and deploying equitable models of eye health delivery [36, 37].

Several limitations of the present study should be noted. The cross-sectional nature precluded conclusions about causation, given the possibility of reverse causality. The individuals who did not complete both visual acuity screening and the questionnaire may have differed systematically and resulted in selection bias, although we found no evidence of a difference in age or sex between the two groups. SES was calculated at the household level and assumed to apply to all household members equally. Ordinal data on visual acuity were dichotomized, which reduced statistical power but made for more easily interpretable regression models. The relatively small sample size increased the uncertainty of prevalence estimates. The small number of blind individuals and their uneven distribution across the socioeconomic groups prevented a separate analysis looking exclusively at the relationship between SES and blindness. While the sampling schema for the parent trachoma study increased the study’s generalizability within this region of Peru, the generalizability of the findings outside of Alto Amazonas is not clear; it is possible that SES has a weaker association with VI and blindness in other locations with better access to eye health services.

In summary, we found that belonging to a higher SES group resulted in a lower odds of VI or blindness compared to those in the lowest group, regardless of the manner in which the groups were constructed. The observation of a linear dose response provides confidence in the observed association, but reverse causality remains a concern. Although studies in other areas of the Amazon basin would be helpful to assess generalizability, these findings can aid public health planners identify at-risk groups who would benefit the most from ocular health interventions.

## Summary

### What was known before

- SES has been associated with various adverse visual outcomes. This relationship is accentuated in rural areas. To date there exist little data from the Amazon basin.

### What this study adds

- This study reports the relationship between VI or blindness and SES in the Peruvian Amazon. It was found that lower SES was associated with higher odds of VI or blindness.

## Supplementary information

Supplemental figure and table legends

Supplemental Figure 1

Supplemental Figure 2

Supplemental Table 1

## Data Availability

The data that support the findings of this study are available on request from the corresponding author. The data are not publicly available due to their containing information that could compromise the privacy of research participants.

## References

[1] Bourne RRA, Flaxman SR, Braithwaite T, Cicinelli MV, Das A, Jonas JB (2017). Magnitude, temporal trends, and projections of the global prevalence of blindness and distance and near vision impairment: a systematic review and meta-analysis. Lancet Glob Health.

[2] Bourne RRA, Stevens GA, White RA, Smith JL, Flaxman SR, Price H (2013). Causes of vision loss worldwide, 1990–2010: a systematic analysis. Lancet Glob Health.

[3] Zheng Y, Lamoureux E, Finkelstein E, Wu R, Lavanya R, Chua D (2011). Independent impact of area-level socioeconomic measures on visual impairment. Investig Ophthalmol Vis Sci.

[4] Rius A, Guisasola L, Sabidó M, Leasher JL, Moriña D, Villalobos A (2014). Prevalence of visual impairment in El Salvador: inequalities in educational level and occupational status. Rev Panam Salud Publica.

[5] Yan X, Chen L, Yan H (2019). Socio-economic status, visual impairment and the mediating role of lifestyles in developed rural areas of China. PLoS ONE.

[6] Jadoon MZ, Dineen B, Bourne RR, Shah SP, Khan MA, Johnson GJ (2006). Prevalence of blindness and visual impairment in Pakistan: the Pakistan National Blindness and Visual Impairment Survey. Investig Ophthalmol Vis Sci.

[7] Watanabe SES, Berezovsky A, Furtado JM, Kimie Higashi Mitsuhiro MR, Cypel M, Cohen MJ (2019). Population-based cataract surgery complications and their impact on visual status in the Brazilian Amazon region. Am J Ophthalmol.

[8] Cunha CC, Berezovsky A, Furtado JM, Ferraz NN, Fernandes AG, Muñoz S (2018). Presbyopia and ocular conditions causing near vision impairment in older adults from the Brazilian Amazon region. Am J Ophthalmol.

[9] Furtado JM, Berezovsky A, Ferraz NN, Muñoz S, Fernandes AG, Watanabe SS (2019). Prevalence and causes of visual impairment and blindness in adults aged 45 years and older from parintins: the Brazilian Amazon Region Eye Survey. Ophthalmic Epidemiol.

[10] INEI. Definitive results of 2017 National Census. INEI. https://www.inei.gob.pe/media/MenuRecursivo/publicaciones_digitales/Est/Lib1561/ 2017.

[11] Instituto Nacional de Estadística e Informática. Mapa de pobreza monetaria provincial y distrital 2018. Lima, Perú: Instituto Nacional de Estadística e Informática; 2020.

[12] OpenStreetMap Wiki contributors. Key: place. OpenStreetMap Wiki; 2019. https://wiki.openstreetmap.org/w/index.php?title=Key:place&oldid=1910053.

[13] Solomon, Anthony W, World Health Organization, International Trachoma Initiative. Trachoma control: a guide for programme managers. Switzerland: WHO; 2006.

[14] Nesemann J, Muñoz MB, Morocho-Alburqueque N, Quincho-Lopez A, Wittberg DM, Honorio H (2021). Prevalence of trachoma in Alto Amazonas, Loreto Department, Perú. Investig Ophthalmol Vis Sci.

[15] Instituto Nacional de EstadÌstica e Informática Perú. Perú Encuesta Demográfica y de Salud Familiar—ENDES 2012. Lima, Perú: INEI; 2013.

[16] McKenzie D (2005). Measuring inequality with asset indicators. J Popul Econ.

[17] Rutstein SO. The DHS wealth index: approaches for rural and urban areas. Calverton, Maryland, USA: Macro International; 2008.

[18] WHO. International classification of diseases for mortality and morbidity statistics (11th Revision). WHO; 2020. https://icd.who.int/en/.

[19] Vyas S, Kumaranayake L (2006). Constructing socio-economic status indices: how to use principal components analysis. Health Policy Plan.

[20] Beckers J-M, Rixen M (2003). EOF calculations and data filling from incomplete oceanographic datasets. J Atmos Ocean Technol.

[21] Barros AJ, Hirakata VN (2003). Alternatives for logistic regression in cross-sectional studies: an empirical comparison of models that directly estimate the prevalence ratio. BMC Med Res Methodol.

[22] Filmer D, Pritchett LH (2001). Estimating wealth effects without expenditure data-or tears: an application to educational enrollments in states of India. Demography.

[23] Gwatkin DR, Rutstein S, Johnson K, Suliman E, Wagstaff A, Amouzou A. Socio-economic differences in health, nutrition, and population: Nigeria 1990, 2003. Country reports on HNP and poverty. Washington, DC: World Bank; 2007. 18293634

[24] Pongo Aguila L, Carrión R, Luna W, Silva JC, Limburg H (2005). Cataract blindness in people 50 years old or older in a semirural area of northern Peru. Rev Panam Salud Publica.

[25] Campos B, Cerrate A, Montjoy E, Dulanto Gomero V, Gonzales C, Tecse A (2014). National survey on the prevalence and causes of blindness in Peru. Rev Panam Salud Publica.

[26] Duerksen R, Limburg H, Carron JE, Foster A (2003). Cataract blindness in Paraguay-results of a national survey. Ophthalmic Epidemiol.

[27] Beltranena F, Casasola K, Silva JC, Limburg H (2007). Cataract blindness in 4 regions of Guatemala: results of a population-based survey. Ophthalmology.

[28] Nano ME, Nano HD, Mugica JM, Silva JC, Montaña G, Limburg H (2006). Rapid assessment of visual impairment due to cataract and cataract surgical services in urban Argentina. Ophthalmic Epidemiol.

[29] Siso F, Esche G, Limburg H. Test nacional de catarata y servicios quirúrgicos “RACSS rapid assesment of cataract and surgical services” primera encuesta nacional. Rev oftalmol venez. 2005;61:112–39.

[30] Limburg H, von-Bischhoffshausen FB, Gomez P, Silva JC, Foster A (2008). Review of recent surveys on blindness and visual impairment in Latin America. Br J Ophthalmol.

[31] Barría F, Silva J, Limburg H, Muñoz D, Castillo L, Martínez L (2008). Análisis de la prevalencia de ceguera y sus causas, determinados mediante encuesta rápida de ceguera evitable (RAAB) en la VIII región, Chile. Chile Arch Chil Oftalmol.

[32] Hernández Silva JR, Río Torres M.Padilla González CM, Resultados del RACSS en Ciudad de La Habana, Cuba, 2005. Rev Cubana Oftalmolía. 2006;19:1–9.

[33] Leasher JL, Lansingh V, Flaxman SR, Jonas JB, Keeffe J, Naidoo K (2014). Prevalence and causes of vision loss in Latin America and the Caribbean: 1990-2010. Br J Ophthalmol.

[34] Silva JC, Mújica OJ, Vega E, Barcelo A, Lansingh VC, McLeod J (2015). A comparative assessment of avoidable blindness and visual impairment in seven Latin American countries: prevalence, coverage, and inequality. Rev Panam Salud Publica.

[35] Salomão SR, Berezovsky A, Furtado JM, Fernandes AG, Muñoz S, Cavascan NN (2018). Vision status in older adults: the Brazilian Amazon Region Eye Survey. Sci Rep.

[36] Natchiar G, Robin AL, Thulasiraj RD, Krishnaswamy S (1994). Attacking the backlog of India’s curable blind. The Aravind Eye Hospital model. Arch Ophthalmol.

[37] Ramke J, Petkovic J, Welch V, Blignault I, Gilbert C, Blanchet K (2017). Interventions to improve access to cataract surgical services and their impact on equity in low- and middle-income countries. Cochrane Database Syst Rev.

