# Supplementary material for: Association of vision impairment and blindness with socioeconomic status in adults 50 years and older from Alto Amazonas, Peru
Source: Eye (Lond). 2022 Feb 3;37(3):434–9. doi: 10.1038/s41433-021-01870-x (PMC9905540; doi:10.1038/s41433-021-01870-x)
Supplement: Supplementary file 1 — Supplemental figure and table legends [file 41433_2021_1870_MOESM1_ESM.docx]

**Supplemental Figure 1. Map of Perú indicating the location of Alto Amazonas.** The inset in the upper left shows the study location within Peru. The solid yellow line represents roads while the pale dotted lines represent dirt paths. Study villages, other villages, and optometrist and ophthalmologist offices are demarcated in black, grey, and red, respectively.

**Supplemental Figure 2. Peruvian Ministry of Health 3-meter visual acuity card.**

Supplemental Table 1. Number of responses, mean, and standard deviation of each socioeconomic variable collected during the study.
