# Supplementary figures and images for: Association of vision impairment and blindness with socioeconomic status in adults 50 years and older from Alto Amazonas, Peru
Source: Eye (Lond). 2022 Feb 3;37(3):434–9. doi: 10.1038/s41433-021-01870-x (PMC9905540; doi:10.1038/s41433-021-01870-x)

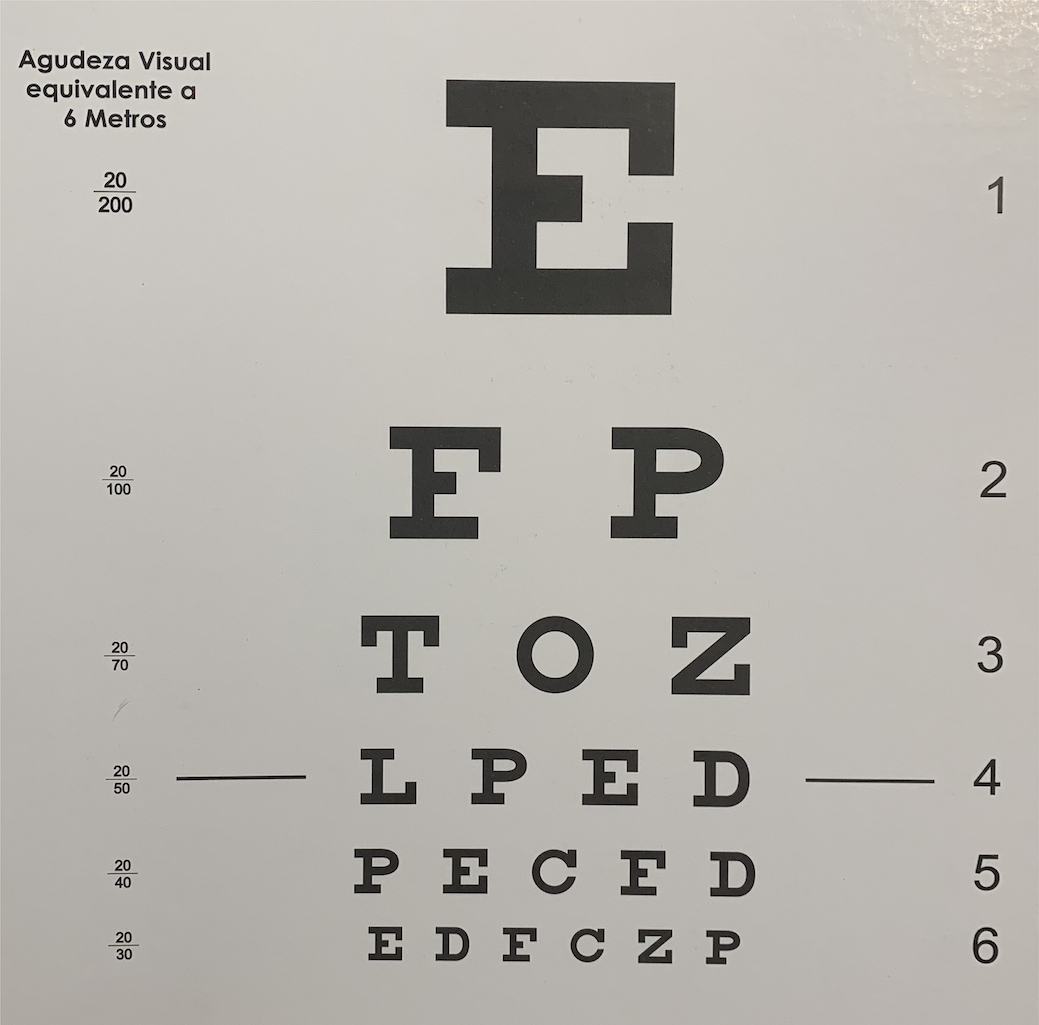

Supplement: Supplementary file 3 — Supplemental Figure 2 [file 41433_2021_1870_MOESM3_ESM.jpg]
