# Supplementary material for: Association of vision impairment and blindness with socioeconomic status in adults 50 years and older from Alto Amazonas, Peru
Source: Eye (Lond). 2022 Feb 3;37(3):434–9. doi: 10.1038/s41433-021-01870-x (PMC9905540; doi:10.1038/s41433-021-01870-x)
Supplement: Supplementary file 4 — Supplemental Table 1 [file 41433_2021_1870_MOESM4_ESM.docx]

Supplemental Table 1. Number of responses, mean, and standard deviation of each socioeconomic variable collected during the study.

| **Variable** | **Number of Responses (%) N=146** | **Mean** | **Standard Deviation** |
| --- | --- | --- | --- |
| **Quantitative variables** | | | |
| Number of hectares owned | 135 (92.5%) | 1.489 | 1.292 |
| Number of birds owned | 142 (97.3%) | 13.049 | 11.341 |
| Number of rooms in the household | 142 (97.3%) | 2.401 | 0.826 |
| **Dichotomous variables ^a^** | | | |
| Guinea pigs owned* | 139 (95.2%) | 0 | 0 |
| Pigs owned | 133 (91.1%) | 0.03 | 0.171 |
| Cows owned | 130 (89%) | 0.031 | 0.173 |
| Unpaid housework | 142 (97.3%) | 0.014 | 0.118 |
| Payed housework | 142 (97.3%) | 0.021 | 0.144 |
| Intellectual work | 142 (97.3%) | 0.028 | 0.166 |
| Non-agricultural work | 142 (97.3%) | 0.028 | 0.166 |
| Self employed | 142 (97.3%) | 0.028 | 0.166 |
| Unemployed | 142 (97.3%) | 0.077 | 0.268 |
| Fisherman | 142 (97.3%) | 0.085 | 0.279 |
| Agricultural worker | 142 (97.3%) | 0.718 | 0.451 |
| Blender owned | 139 (95.2%) | 0.007 | 0.085 |
| Motorcycle owned | 133 (91.1%) | 0.015 | 0.122 |
| Cook with natural gas | 142 (97.3%) | 0.021 | 0.144 |
| Dresser owned | 140 (95.9%) | 0.021 | 0.145 |
| Cellphone owned | 142 (97.3%) | 0.134 | 0.342 |
| Radio owned | 137 (93.8%) | 0.292 | 0.456 |
| Solar panel owned | 133 (91.1%) | 0.316 | 0.467 |
| Hammock owned | 137 (93.8%) | 0.759 | 0.429 |
| Cook with wood | 142 (97.3%) | 0.979 | 0.144 |
| Dirt floor | 142 (97.3%) | 0.282 | 0.451 |
| House has windows | 140 (95.9%) | 0.471 | 0.501 |
| Wooden floor | 142 (97.3%) | 0.718 | 0.451 |
| Flush to septic tank | 142 (97.3%) | 0.007 | 0.084 |
| Covered pit latrine | 142 (97.3%) | 0.007 | 0.084 |
| Flush to drain | 142 (97.3%) | 0.014 | 0.118 |
| Ventilation improved pit latrine | 142 (97.3%) | 0.021 | 0.144 |
| Flush to pit latrine | 142 (97.3%) | 0.028 | 0.166 |
| Open pit latrine | 142 (97.3%) | 0.169 | 0.376 |
| Handwash station | 142 (97.3%) | 0.282 | 0.451 |
| No latrine (open defecation) | 142 (97.3%) | 0.754 | 0.432 |
| Protected spring | 144 (98.6%) | 0.007 | 0.083 |
| Unprotected well | 144 (98.6%) | 0.007 | 0.083 |
| Piped into dwelling | 144 (98.6%) | 0.028 | 0.165 |
| Protected well | 144 (98.6%) | 0.042 | 0.201 |
| Unprotected spring | 144 (98.6%) | 0.062 | 0.243 |
| Surface water | 144 (98.6%) | 0.854 | 0.354 |

* These variables were omitted in the construction of the SES index. ^a^ The mean for dichotomous variables provides the proportion answering affirmatively to the question
